# Announcing the *International Journal of Molecular Sciences* Junior Scientists Travel Awards 2016

**DOI:** 10.3390/ijms17030405

**Published:** 2016-03-19

**Authors:** International Journal of Molecular Sciences Editorial Office

With the goal of recognizing outstanding contributions to the field of molecular sciences by early-career investigators, including assistant professors, postdoctoral students and PhD students, and assisting them in attending international conferences in 2016, last year the *International Journal of Molecular Sciences* accepted nominations for Junior Scientists Travel Awards 2016. Over 300 nominations were received and were evaluated by a panel of judges comprised of *International Journal of Molecular Sciences* editorial board members.

We are excited to announce the following winners: Dr. Bert De Rybel, Dr. Jean Christopher Chamcheu, Dr. Jean-Marie Swiecicki, Mr. Pedro Rodrigues, and Dr. Renato Polimanti. They will be supported with up to 800 Swiss Francs each towards their travel expenses to attend international conferences in 2016.

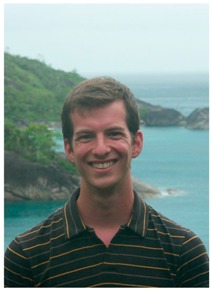

Dr. Bert De Rybel

Dr. Bert De Rybel, Project Leader at VIB-Ghent University, Belgium. Dr. De Rybel completed his Ph.D. degree in the Department of Plant Systems Biology (VIB-Ghent University, Belgium), supervised by Professor Tom Beeckman, focusing on early lateral root development. This resulted in publications in several leading journals such as *Science*, *Nature*, *Nature Chemical Biology* and *Current Biology*. He then moved to the laboratory of Professor Dolf Weijers in Wageningen (the Netherlands), funded by FEBS and Marie Curie post-doctoral fellowships, to work on embryo development and initiated work into vascular development. After five years of post-doctoral research in which he published his work in leading journals including *Science* and *Developmental Cell*, Dr. De Rybel received an FWO Odysseus return grant to set up his own line of research at the department of Plant Systems Biology in VIB-Ghent University, Belgium, where he currently works as a project leader on vascular development in plants. At the same time, he remains associated to Wageningen University, funded by an NWO VIDI grant. His current research focuses on understanding how plant vascular cells control the orientation of their cell divisions and how this has developed through evolution. When not in the lab, Dr. De Rybel enjoys Belgian beers, painting and hiking/camping.

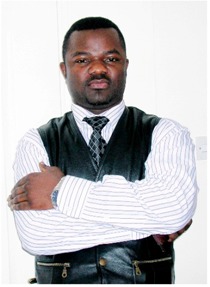

Dr. Jean Christopher Chamcheu

Dr. Jean Christopher Chamcheu, Assistant Scientist at University of Wisconsin (UW)-Madison, Madison, WI, USA. Dr. Chamcheu earned his BS degree in Biochemistry with a minor in Biomedical Analyses and Pharmacology at the University of Dschang, Cameroon, and his MS degree in Biomedicine (2004), at Linkoping University in Sweden. He completed his Ph.D. degree in Dermatology and Venereology (2010) at Uppsala University in Sweden, working with Professors Hans Torma and Anders Vahlquist, to identify the underlying molecular genetic basis and develop new pharmacologic strategies for treating epidermolytic keratinopathic genodermatoses. On completion of his Ph.D., Dr. Chamcheu joined the Department of Dermatology at the UW-Madison as a Postdoctoral Research Associate, working with Professor Hasan Mukhtar for over 3.5 years. His initial research focused on defining the role of Caspase-14 in the pathogenesis of psoriasis, a chronic immune-mediated inflammatory skin disease, and in establishing the utility of delphinidin, a novel small molecule for treating psoriasis. In 2014, he was promoted to the scientist track, where he currently works as an Assistant Scientist, a position at UW-Madison (equivalent to the rank of Research Assistant Professor). Dr. Chamcheu is now developing his research program in psoriasis with studies aimed at uncovering the underlying and regulatory mechanisms and contribution of PI3K/Akt/mTOR signaling to psoriasis pathogenesis, and its targeting, using natural small molecule inhibitors with the hope to identify novel prognostic markers and therapeutic targets for treating psoriasis and possibly other hyperproliferative and inflammatory skin disorders. Dr. Chamcheu has recently received a number of grants and awards as co-investigator or as principal investigator, including an American Skin Association Carson Research Scholar Award in Psoriasis. When Dr. Chamcheu is not in the lab, he enjoys outdoor activities, such as playing football soccer, and participates in community outreach organizations, such as the Powerman HOPE Foundation International.

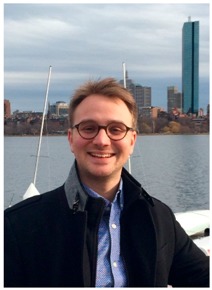

Dr. Jean-Marie Swiecicki

Dr. Jean-Marie Swiecicki, Post-Doctoral Research Associate at Massachusetts Institute of Technology, USA. Dr. Swiecicki completed his undergraduate studies in 2011 at the École Normale Supérieure in Paris with a major in Chemistry and a minor in Biology. He then obtained his Ph.D. degree from the University Pierre and Marie Curie in 2014 working under the guidance of Professor Solange Lavielle. His graduate research focused on the development of original strategies to interrogate cellular internalization mechanisms and the subsequent intracellular distribution of cell-penetrating peptides. His studies provided a comprehensive dynamic picture of the internalization processes, which he then used to rationally design a new delivery vector. Continuing on his academic path to decipher the elementary principles of membrane-associated processes, he is now working in Prof. Barbara Imperiali’s laboratory at MIT. As a post-doctoral associate, he is developing an integrated platform for characterizing molecular interactions among associated peripheral and integral membrane proteins, as well as the membrane lipids environment. He is currently applying this platform to the membrane-bound enzymes of the *N*-linked protein glycosylation pathway of the human pathogen bacterium, *Campylobacter jejuni*. When not in the lab, Dr. Swiecicki enjoys baking at home, sailing on the Charles River and discussing politics.

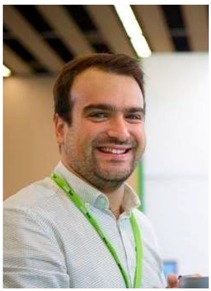

Mr. Pedro Rodrigues

Mr. Pedro Rodrigues, Ph.D. Student at Research Institute for Medicines (iMed. ULisboa), Portugal. Pedro received his BS degree in Molecular and Cellular Biology from the New University of Lisbon in 2011 and a post-graduation in Biopharmaceutical Sciences in 2012. Since beginning his Ph.D. studies in 2013, Pedro has investigated the role of microRNAs and nuclear receptors in non-alcoholic fatty liver disease (NAFLD) pathogenesis, under the supervision of Dr. Rui Castro and Prof. Cecília Rodrigues. NAFLD can lead to cirrhosis and hepatocellular carcinoma which lack pharmacological therapeutic options. Studies performed by Pedro have already highlighted novel molecular therapeutic targets that may be useful in developing new drugs to treat NAFLD. He has presented his work in several national and international conferences and is the author of several scientific papers. Pedro has also received several grants and honors, including a National Scholar Award at UEG Week 2014, and his work has been distinguished at several conferences. Outside the lab, Pedro likes to play the clarinet and is a member of several bands and orchestras in Lisbon and in his hometown, Tomar.

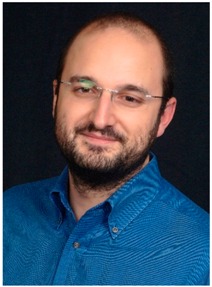

Dr. Renato Polimanti

Dr. Renato Polimanti, Post-Doctoral Research Associate at Yale University School of Medicine and VA CT Healthcare Center, USA. Dr. Polimanti received his Ph.D. degree from the University of Rome “Tor Vergata”, Italy, where he studied the evolution of pharmacogenetic systems among human populations. Since 2013, he has been a postdoctoral associate in the group of Dr. Joel Gelernter in the Division of Human Genetics of the Department of Psychiatry at Yale University School of Medicine. Dr. Polimanti’s research is currently focused on the understanding of the pathogenic mechanisms of traits related to alcohol and drug use disorders using various types of genomic data. His recent studies provided novel findings about the role of ancestry genomic background in genome-wide association studies of substance dependencies (Polimanti *et al*., 2015, *Pharmacogenomics*) and the molecular mechanisms by which alcohol dependence affects body mass regulation (Polimanti *et al*., 2015, *Addiction Biology*). He is an author of over 50 scientific articles published in international peer-reviewed journals. Dr. Polimanti has received several honors and awards including the NARSAD Young Investigator Award from Brain and Behavior Research Foundation and the Early-Career Investigator Travel Award from the Society of Biological Psychiatry.

On behalf of the *International Journal of Molecular Sciences’* editorial board members and editorial staffs, we wish to congratulate these five outstanding junior scientists for their accomplishments.

